# A Comparative Study of the Role of Australia and New Zealand in Sustainable Dairy Competition in the Chinese Market after the Dairy Safety Scandals

**DOI:** 10.3390/ijerph15122880

**Published:** 2018-12-15

**Authors:** Junqian Xu, Yuanyuan Wu

**Affiliations:** School of Business, Jiangnan University, Wuxi 214122, China; jxu2000@jiangnan.edu.cn

**Keywords:** dairy imports, China, Australia, New Zealand, melamine milk scandal

## Abstract

After the melamine milk scandal in 2008, China’s global imports of dairy products soared, especially after FTAs had been established with Australia and New Zealand. The dairy products of the two countries have a unique competitive trading advantage in the Chinese market. However, at a time when Chinese consumers are increasingly dependent on imported dairy products, a succession of whey protein scandals affecting New Zealand’s dairy products in 2013 had a negative psychological impact on Chinese importers and consumers, and this even affected the import status of New Zealand dairy imports to the Chinese market. The present paper, based on the United Nations Comtrade Harmonized System, studies the role of Australia and New Zealand in China’s dairy market. It calculates the trade competitiveness index, revealing the relative competitive advantages of Australia and New Zealand, and investigates the impact of the dairy products from these countries on China’s imports from the rest of the world across six dairy sectors in the period 1992–2017. We find that, under the food safety laws, the relative dairy import prices, milk scandals, and Free Trade Agreements, together with the competitive advantages of Australia and New Zealand, had a varied impact on the corresponding Chinese dairy imports across the relevant sectors in the context of China food safety laws after the melamine milk scandal. These findings acknowledge Australia and New Zealand’s competitiveness in the international dairy trade, and also lead to suggestions regarding their competitiveness and sustainable development in the Chinese market.

## 1. Introduction

China, one of the largest dairy consuming countries, has increased its consumption rapidly since the 1990s. China’s entry into the WTO in 2001 had a huge impact on its own dairy trade. China’s domestic dairy industry was developing rapidly; the domestic demand for dairy products had increased and even exceeded supply, with diverse levels of consumption. All this made China’s domestic market competition extremely fierce. During this time, the price forming mechanism of China’s dairy industry chain was imperfect, the benefit distribution of its dairy industry chain was distorted [1,2,3,4,5,6] and a series of dairy product safety scandals shook the confidence of Chinese consumers. Of all the dairy scandals, the 2008 melamine milk scandal had the biggest impact on the dairy industry. Many families suffered heavy losses and consumers lost trust in the Sanlu company, which in turn damaged the reputation of the Chinese dairy industry as a whole [7,8]. China’s dairy imports are still on the rise and now New Zealand has become the largest source of its dairy imports [9].

The China melamine milk scandal in 2008 exposed the weak points of many important production links. In the link of between collecting the raw milk, transporting, and storing it, milk collection stations play an important part [10,11,12]. In China, individual milk collection stations are in a dominant position [13]. These numerous small stations are non-standardized and difficult to supervise. At the same time, regulatory departments are independent and have overlapping duties, making cooperation and coordination difficult. This results in conflicts between milk stations and regulators over functions and power and has also led to a lack of supervision in some links of the dairy chain [14,15]. The Chinese government has issued a succession of laws and regulations to tighten milk quality control, notably, the Food Safety Law of the People’s Republic of China, which came into effect in June 2009. This symbolized that the food safety supervision model in China had moved to a transitional and reformatory stage, where it changed from multi-agency coordination to centralization [16].

However, although the Chinese government has begun to monitor strictly the quality of dairy products, domestic enterprises have increased the imports of dairy-related materials. The import of dairy products increased rapidly, and the dairy trade remained in deficit, so China is heavily reliant on the international market. Since China’s entry into the WTO, more than half of its imports of dairy products have come from Australia and New Zealand. After the melamine milk scandal, China’s dairy imports from Australia and New Zealand rose sharply, to over 70 percent of its total imports in 2010 [17]. China’s dairy scandal has had a significant impact on China’s overall trade and since China is one of the largest dairy importers in the world, the impact even affects the world dairy market. At a time when Chinese consumers were in a crisis of trust regarding the domestic dairy industry, China was also tightening its quality checks on dairy production and imports.

In 2013, New Zealand, China’s largest source of milk powder, detected abnormal levels of nitrate in whey protein three times in the same month, at a time when 390 kg of products had been exported to China for the production of dairy consumables. China’s General Administration of Quality Supervision, Inspection and Quarantine (AQSIQ) immediately suspended imports of lactoferrin from the Westland company of New Zealand and required nitrate testing reports whenever lactoferrin was imported from other New Zealand companies and whenever other dairy products were imported from Westland. In the same year, the New Zealand dairy giant Fonterra found some of its whey protein contaminated by Botox, prompting a number of well-known milk powder companies to recall products that contained this ingredient. Fonterra promptly apologized to Chinese consumers for the quality of its dairy products, and the Prime Minister of New Zealand condemned the incident, while its government has constantly sought to verify the quality of dairy products made by this company. These actions quickly restored the reputation of New Zealand’s dairy products and re-established the trust of Chinese consumers.

**Figure 1:**
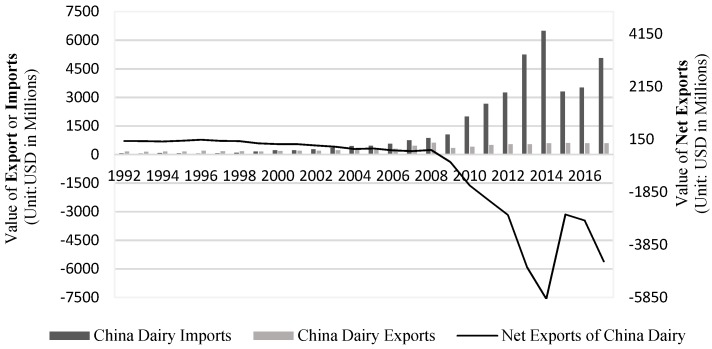
China Dairy Exports and Imports from World (Unit: USD in Millions).

China’s growth of dairy exports has been stable since the 1990s but is dwarfed by imports (shown in Table 1). China’s dairy imports have grown rapidly and risen sharply since 2008. For example, the demand for dairy imports in 2017 was eight times higher than in 2008. Hence, China’s dairy deficit, $251.8 million in 2008, began to widen, reaching $4.4817 billion in 2017. Figure 1 shows that China’s trade deficit suddenly increased after 2008, but the trend eased slightly in 2014. Due to the Fonterra milk scandal, China stopped importing whey protein products from New Zealand in August 2013, which also dampened China’s overall imports of dairy products in 2013 and 2014. However, because New Zealand’s reputation recovered so fast, there was a sharp increase in overall imports, even after a decline in 2015 in all China’s dairy imports and a declining trend in New Zealand’s market share in China. Table 2 shows that China’s imports of all dairy products dramatically increased after 2008, especially solid dairy product sectors such as HS0402, HS0404 and HS0405. Imports in all sectors climbed beyond those in 2015. China’s trade demand from all the sectors of dairy products shows that it had the highest demand for concentrated milk (sector 2), followed by whey (sector 4), and not concentrated milk (sector 1 after 2015). The demand for cheese and curd (sector 6) and butter (sector 5) also soared.

According to the 2017 Dairy Industry Yearbook of China [18], China’s imports of dairy products in 2016 increased by 13.5% in the following year. Among them, imports of liquid milk (including liquid milk HS0401 and yoghurt HS0403) increased by 38.8%; imports of solid dairy products (at HS0402, HS0404, HS0405, HS0406) increased by 13.2%. In 2017, China imported 11.912 million tons of raw milk while its domestic fresh milk production fell and the self-sufficiency rate of its dairy production fell to 75.5%. There are many reasons for the increase in China’s dairy imports, such as the fall in the prices charged by major international milk production countries, the higher cost of major agricultural products in China compared with their imported equivalents, and the rising costs of production. The melamine milk scandal in 2008 was related to the impact of imported milk powder and the fall in raw material prices. At the same time, when China’s melamine milk scandal occurred in 2008, falling prices in response to the 2008–2009 financial crisis in major dairy production areas such as New Zealand and Australia had accelerated the flow of foreign milk powder to the international market where demand was greatest, China in particular. In addition, the high subsidies in developed countries have further enhanced the price advantage of their dairy products [19].

The European Union announced export subsidies for dairy products in 2009 [20], and the United States proposed export subsidies in the same year. Australia, New Zealand and other countries have also introduced policies in order to increase export competitiveness in the global market [21]. China’s demand for dairy imports has remained consistently high in recent years. The establishment of dairy trade agreements with New Zealand and Australia means that more than half of China’s dairy import tariffs from world sources have been further reduced, which has had a huge impact on China’s dairy industry and import demand [22].

The melamine milk scandal hugely increased China’s demand for imported dairy products and has even had a direct impact on the relationship between supply and demand in the international dairy market which may have far-reaching implications for Australia and New Zealand, as major dairy exporting countries, in the international market. In recent years, much scholarly attention and research has been devoted to the dairy scandal in China [16,23,24,25], but there has been little comparative analysis between Australia and New Zealand to assess the relative global impact of dairy imports on the Chinese market. The present research will compare the performance of China’s global dairy imports before and after entry into the WTO in the period of 1992–2017, across all sectors in relation to Australia and New Zealand especially in the context of the food safety laws following the milk scandals. The underlying purpose is to investigate the international roles of New Zealand and Australia in the Chinese dairy import market.

The remaining sections in this paper are as follows: Section 2 examines Australia and New Zealand’s share in the Chinese dairy product market at different time periods; Section 3 compares the competitiveness of Australia and New Zealand in this market; Section 4 and Section 5 contain the model specification and econometric modeling as well as a classification of the data used in the study; Section 6 analyzes the influence of Australia and New Zealand on China’s dairy import market; and Section 7 draws some conclusions.

## 2. Comparison of the Dairy Shares of Australia and New Zealand in the Chinese Market

Australia and New Zealand have long been China’s leading source of dairy products, and their market share in China has risen to more than 60 percent following the Chinese melamine milk scandal. So far, New Zealand is still China’s largest source of dairy products with a considerable competitive advantage. At present, New Zealand is the largest supplier of formula milk imported to China. Livestock is the main source of income, and the New Zealand government tests and monitors dairy animals very strictly. New Zealand is famous for its clean air and green ecology; all its cows are free-range and enjoy natural fodder. Antibiotics are not used to treat dairy cows and nor are hormones are used to increase yields. The production of milk powder is based on the advanced wet process, which preserves its nutritional value and reduces the risk of secondary pollution [27]. The expansion of New Zealand’s dairy market share in China increased strongly in the wake of the melamine scandal in 2008, the year when the China—New Zealand free trade agreement came into effect—China’s first Free Trade agreement with a developed country [28]. Among the important terms of the agreement were arrangements for tax reduction and a special safeguard mechanism for dairy products. As the world’s first country to export dairy products, New Zealand produces them on a large scale, and has great potential for increasing production and an obvious international competitive advantage [29,30]. New Zealand has actively expanded its dairy exports to China through the construction of a free trade zone, and the dairy trade has achieved zero tariffs. The duty-paid price of New Zealand milk powder is about 1000 US dollars per ton cheaper than that of EU milk powder, and the cost of exporting to China has been reduced by an average of 13.4 percent, which gives New Zealand dairy products an overwhelming advantage in the Chinese market, making China also the largest export market for New Zealand dairy products [22,31]. It also deepens the influence of New Zealand dairy products on the balance of supply and demand in the Chinese dairy market [28]. Unfortunately, a series of whey protein scandals of New Zealand in 2013 has alerted China to the need to focus on other sources of milk. Australia thus became an ideal choice.

Like New Zealand, Australia is surrounded by the sea, with a beautiful natural environment, developed agriculture and a large supply of milk. The Australian government has strict regulatory conditions that make the milk source safe: dairy cattle develop without antibiotics, feeding on green plants and safe grasses free from pesticides. On 17 June 2015, China and Australia formally signed a “Free Trade Agreement between the Government of the People’s Republic of China and the Government of Australia” [32], indicating that Australia would also become one of the most important sources of dairy imports for China after New Zealand. The Agreement has played a positive role in diversifying China’s dairy imports and avoiding excessive reliance on a single market. Australia is a world-famous dairy country: the average annual output of its dairy industry is about 13 billion Australian dollars. Although Australia accounts for only 2% of the world’s milk production, its exports account for about 7% of the world’s dairy trade, second only to New Zealand and the European Union, and the third largest exporter of dairy products in the world [32].

China’s signing the FTAs with New Zealand and Australia consolidated their bilateral trade and international cooperation, to the benefit of the domestic consumers of China [33]. New Zealand and Australia have become staple dairy suppliers to China, and thereby have taken an important position in the world dairy trade [28,32]. International market share is the most direct and simple indicator of this, reflecting the international competitiveness or competitive status of a country’s industry or product. The higher the market share, the stronger the international competitiveness; conversely, the lower the market share, the weaker the international competitiveness [34]. On the proportion of imports from China’s dairy products market (shown in Table 3), after 2000, the ratio of Chinese imports from New Zealand and Australia has consistently exceeded 50, reaching 72.52 in 2010, though it fell after the New Zealand dairy scandal. But China’s share of dairy imports from Australia and New Zealand as two major free trade zones in 2017 still stood at 66.80.

Table 2 shows steady growth in all sectors of dairy imports from New Zealand, notably Sector 2 and Sector 4. Nevertheless, Table 4 reflects a steady increase in the share of New Zealand dairy products in the Chinese market. But after the whey scandal in 2013, the share of the two major export sectors in the Chinese market fell, and both fell back to 2000 levels in 2015. In particular, the share of Sector 4, whey protein, in the Chinese market fell to 0.44% in 2015, lower than 0.47% in 2000. The continued decline in China’s market share in 2017 reflects, indirectly, the weakening competitiveness of New Zealand dairy products in the Chinese market following the whey protein scandal. Compared with New Zealand, the Chinese market share of Australian dairy raw materials in the early 1990s was mainly in Sectors 1 and 6, both higher than New Zealand, indicating that Australian competitiveness was stronger. But with China’s accession to the WTO, China imported more dairy products from New Zealand. We also observed that the market share of Australian dairy products in China was basically stable, and also was affected by the New Zealand whey protein scandal, China’s dairy imports from Australia in Sectors 2 and 4 also showed a downward trend, but the market share was not as volatile as New Zealand’s share.

## 3. Trade Competitiveness of Australia and New Zealand in the Chinese market

New Zealand has always advocated the production of 100% pure natural dairy products, and its trade competition index (TC) is the highest in the world, almost always at the level of 1, and only below it for very few years [28]. Table 5 shows that New Zealand has a higher overall competitiveness index in dairy products (HS04) than Australia, with a TC at 1 for all sectors since 2000, and close to 1 for only a few years. For example, the TC of concentrated milk and cream containing added sugar >1.5% (HS040229) is 0.996. After the New Zealand whey crisis in 2013, Chinese consumers boycotted the import of New Zealand dairy products. Nevertheless, due to New Zealand’s positive response measures, the figure for its HS0404 division’s competitiveness index in the Chinese market has always remained at 1, indicating that it remains highly competitive.

Meanwhile, Australian dairy products as a whole and several of its sectors were highly competitive in the Chinese market, approaching 1. But compared with New Zealand, with the exception of a TC equal to 1 for butter and other fats and oils derived from milk and dairy spread (at HS0405), other sectors have individual year indices slightly lower than 1. Table 3 shows that New Zealand has the highest share of the Chinese market, and Table 5 further demonstrates that New Zealand dairy industries are the most competitive in the Chinese market. Even though New Zealand’s whey products at sector HS0404 suffered a quality crisis in 2013 and its market share in China fell, the competitiveness index of this sector was still 1, higher than that of Australia, illustrating New Zealand’s dominant position in dairy exports to China.

The revealed comparative advantage index (or RCA) [24,34,35], can help us to measure the international competitiveness of a country’s industry or products. For dairy products as a whole, New Zealand has a greater comparative advantage, a very high degree of specialization and a strong international competitiveness [28]. Table 6 shows that the overall RCA in China of dairy products from New Zealand is higher than those from Australia, especially after 2000, when New Zealand’s RCA was greater than 1, indicating that New Zealand’s dairy products have a strong comparative advantage. Compared with New Zealand, the comparative advantage of the dairy products (HS04) from Australia was altogether inferior. Across sectors, the RCA of Australian liquid dairy goods or non-concentrated milk and cream (HS0401) has improved significantly since 2010, especially for sectoral products with a fat content ≤6% (HS040110 and HS040120), but New Zealand’s comparative advantage in the HS0401 sector seems to have been more competitive in 2010. The RCA index was greater than 1 and is significantly higher than Australia’s, implying that New Zealand liquid dairy goods or non-concentrated milk and cream (HS0401) were more competitive in the Chinese market.

China has a huge demand for concentrated milk (HS0402) (shown in Table 2 and Table 4). We observe that in sector HS0402 (concentrated milk and cream), the RCA in New Zealand was still higher than that in Australia, especially since 2000, when the RCA of New Zealand in China was more than 1, indicating that the comparative advantage of New Zealand was greater than 1, which makes its position as the most important dairy exporter in the world clear. As for the buttermilk, curdled milk and cream and yogurt (sector HS0403), although New Zealand’s RCA was still higher than Australia’s, we find that, except for an RCA greater than 1 for New Zealand in 2015, both Australia and New Zealand had an RCA of less than 1, meaning that, in the Chinese market, the comparative advantage of the two countries in this sector was not strong.

In Table 5, we observe that New Zealand’s trade competitiveness index in whey and products consisting of natural milk constituents, the HS0404 sector, has remained at 1 in the Chinese market, and Australia’s TC was also basically 1. Before 2005, the Australian RCA index overall in the HS0404 sector was also greater than 1, but its RCA in whey (HS040410) was significantly lower than New Zealand’s. Unfortunately, despite the fact that its RCA index was higher than that of Australia in 2000–2010 and more than 1 before 2013, when New Zealand had a safety problem with its whey protein (HS040410), it even reached 8.245 and 6.323 in 2005 and 2010 respectively, significantly higher than other sector indices, indicating that New Zealand’s whey protein was highly competitive in the Chinese market, second only to its milk powder (Table 2) After the whey protein scandal in 2013, however, New Zealand’s RCA in this sector fell significantly to 0.952 in 2015, leading to an overall decline in its RCA in the HS0404 sector. Nevertheless, New Zealand’s RCA remains higher than Australia’s. From this, we can see New Zealand’s comparative advantage in China. Butter and cheese, in sectors HS0405 and HS0406 respectively are similar to buttermilk in sector HS0403, where the RCA of both New Zealand and Australia was less than 1, showing that the competitive advantages of both countries were not obvious in the Chinese market, though the RCA of New Zealand was slightly higher than that of Australia.

In summary, through the analysis of their international market shares, competitiveness indices, and competitive advantage, New Zealand and Australia are very strongly competitive in the Chinese dairy industry. New Zealand’s comparative advantage in the Chinese market was more evident, especially in the two major sectors of HS0401, HS0402. New Zealand whey protein was highly competitive in the Chinese market before 2013, having the greatest comparative advantage in the HS0404 sector of all, but unfortunately, with the appearance of the whey protein scandal, although the trade competitiveness index of New Zealand’s whey remains at 1, the revealed comparative advantage or RCA of whey plummeted.

## 4. Model Specification

Both Australia and New Zealand have obvious comparative advantages in the Chinese dairy market, and the relative price of their dairy goods may have diverse effects on different sectors of dairy products at different times. As Bini-Smaghi (1991) [36] suggests, different sectors make different import demands and have different price elasticities. A bilateral sectoral study is frequently conducted using data separated into “differentiated” and “commodity” trading [37,38].

The “Theory of Demand for Products Distinguished by Place of Production” has been derived by Armington (1969) [39]. That analysis assumes that consumer utility for goods in an industry is separable from the consumption of other products and imported goods and that their domestic counterparts are incomplete substitutes. Both price and output elasticities can be estimated by the Armington model. Elasticities of substitution between imports and competing domestic products play a key role in open-economy computable general equilibrium (CGE) modeling [38]. Armington (1969) [39] and most CGE modelers have used the constant elasticity of substitution (CES) form for an industry group, where utility is derived from domestic and foreign goods:

(1) $$U={\left[\gamma \;M^{(\theta -1)/\theta }+(1-\gamma )D^{(\theta -1)/\theta }\right]}^{\theta /(\theta -1)}$$

where θ is the constant elasticity of substitution between the domestic and traded goods (Armington elasticity), M is the trade volume, which in the present study is the volume of imported goods, and D is the volume of domestic goods. γ and 1−γ are the distribution parameters associated with *M* and *D* (indices for industry groups are omitted).

Cost minimization subject to the above utility function implies the first-order condition that the marginal rate of substitution between *M* and *D* should equal the corresponding price ratio $\frac{P^{M}}{P^{D}}$. This condition can be solved for the quantity ratio of imported and domestic products in Equation (2) as follows:

(2) $$\frac{M}{D}={\left[(\frac{\gamma }{1-\gamma })\frac{P^{M}}{P^{D}}\right]}^{\theta }$$

where $P^{M}$ and $P^{D}$ are the trade and domestic prices, respectively. Re-writing Equation (2) in logarithmic form, we have:

(3) $$\ln\left[\frac{M}{D}\right]=\theta \ln\left[\frac{\gamma }{1-\gamma }\right]+\theta \ln\left[\frac{P^{M}}{P^{D}}\right]$$

Elasticities are unlikely to be equal across sectors and a simplified form of the level relationship based on bilateral sector data is as follows:

(4) $$\ln M_{ijt}^{China}=\alpha_{0,ij}+\alpha_{1,ij}\ln\frac{P_{ijt}^{M}}{P_{ijt}^{D}}+\alpha_{2,ij}\ln D_{i,t}^{China}+\varepsilon_{ijt}$$

where $\alpha_{1}$ is the price elasticity of substitution. Equation (4) can be estimated within a panel framework, which helps in testing the equivalence of coefficients across sectors: $\alpha_{k,ij}=\alpha_{k,ij}\forall i$. Indeed, in a panel context, we can also combine trade from a number of countries and all sectors in our basic specification: $\alpha_{k,ij}=\alpha_{k,ij}\forall i,j$.

Similarly, China’s imports from Australia or New Zealand (m is Australia or New Zealand) can be written as:

(5) $$\ln M_{i,mt}^{China}=\beta_{0,ij}+\beta_{1,ij}\ln\frac{P_{i,mt}^{M}}{P_{it}^{D}}+\beta_{2,ij}\ln D_{it}^{China}+\varepsilon_{ijt}$$

where $M_{i,mt}^{China}$ is China’s imports from Australia or New Zealand for sector i and $\frac{P_{i,mt}^{M}}{P_{ijt}^{D}}$ is the ratio of the prices of Australia and New Zealand to those of China in sector i.

Taking Equations (4) and (5), the U.S. relative imports can be written as:

(6) $$\ln\frac{M_{ijt}^{China}}{M_{i,mt}^{China}}=\gamma_{ij0}+\gamma_{1,ij}\ln\frac{P_{ijt}^{China}}{P_{i,mt}^{China}}+\varepsilon_{ijt}$$

where $\frac{M_{ijt}^{China}}{M_{i,mt}^{China}}$ is the ratio of China’s imports from country j to China’s imports from Australia or New Zealand in sector i, and $\frac{P_{ijt}^{China}}{P_{i,mt}^{China}}$ is the ratio of the import price of China to that of Australia or New Zealand in sector i. As expected, when the import price from Australia or New Zealand is lower than the others, the relative demand from Australia or New Zealand will increase, so $\gamma_{1,ij}<0$.

In order to have the effects of the dairy safety scandals reflected in China’s dairy imports, three dummies were included in our specification, as follows:

(7) $$\ln\frac{M_{ijt}^{China}}{M_{imt}^{China}}=\gamma_{0,ij}+\gamma_{1,ij}\ln\frac{P_{ijt}^{China}}{P_{imt}^{China}}+\gamma_{2,ij}D_{t}^{WTO}+\gamma_{3,ij}D_{t}^{MMS\_China}+\gamma_{4,ij}D_{t}^{MS\_NZ}\;\;+\gamma_{5,ij}D_{t}^{FTA\_Aus}+\varepsilon_{ijt}$$

where $D_{t}^{WTO}$ means China’s entry into the WTO in 2001. The dummy variable equals 1 for 2001 and after, and 0 before 2001. Since 2001, cooperation between China and abroad has been strengthened and domestic dairy imports have risen rapidly.

$D_{t}^{MMS\_China}$ means China’s melamine milk scandal in 2008. The melamine incident has had a major impact on China’s dairy industry and has had a profound impact on consumers’ willingness to buy. $D_{t}^{MMS\_China}$ is equal to 1 for 2008 and after, and 0 before 2008. In the same year, China signed the FTA with New Zealand.

$D_{t}^{MS\_NZ}$ means whey protein quality incident or milk scandal in New Zealand in 2013. Since it is difficult to capture the influence of scandal, we set this factor as a dummy variable, which equals 1 for 2013 and after, and 0 before 2013.

$D_{t}^{FTA\_Aus}$ means China signed FTA with Australia in 2015 and Australia became one of the most important sources of dairy imports for China after New Zealand.

$D^{FSL\_China}$ means the proxy of the Food Safety Law (FSL) of the People’s Republic of China (Wu et al., 2018), which came into effect in June 2009. The Chinese government issued a series of laws and regulations to enhance milk control afterwards. This paper investigates the role of Australia and New Zealand in China’s dairy market, respectively, under milk scandals from both China and New Zealand in the context of the Food Safety Law of China. The dummy variable equals 1 for 2009 and after, and 0 before 2009.

## 5. The Seemingly Unrelated Regression Model (SUR Model) and Data Classification

We estimate equations across all available sectors of dairy products, based on Equation (7). Ordinary Least Square (OLS) may become an appropriate technique if the residuals across equations are uncorrelated. However, if the residuals are correlated, the equations may be linked. In the equations, all of the variables, such as relative prices, have been used as regressors; thus, the residuals of these equations are subject to cross-correlation, since they are also associated with various import demands for dairy products across the same group of countries.

The seemingly unrelated regression (SUR) model developed by Zellner (1962) [40] is a technique for analyzing a system of multiple equations with cross-equation parameter restrictions and correlated error terms. SUR is an extension of the linear regression model, which allows correlated errors between equations. There are six equations across six sectors of not concentrated milk and cream (HS0401), concentrated milk and cream (HS0402) and cheese and curd (HS0406), respectively; two equations across the two dairy sectors of buttermilk (HS0403) and whey and product consisting of natural milk constituents (HS0404), respectively; and four equations across the four sectors of butter and dairy spread (HS0405). Each equation may satisfy the OLS assumptions, but the joint model exhibits serial correlation due to the correlation of the error terms, and OLS estimation will be inefficient. Using the SUR method to estimate the equations jointly improves efficiency [41].

This paper investigates the role of Australia and New Zealand in China’s relative imports of dairy related products. This analysis covers the bilateral imports of concentrated milk, not concentrated milk, buttermilk, whey and natural milk, butter, cheese and curd between China and the world. Trade data in annual frequencies are taken from the UN Comtrade Database, Harmonized System, which are also categorized into different sectors and listed in Table 7, below as follows.

## 6. The Influence of Australia and New Zealand on China’s Dairy Import Market

The set of results for the impact of relative price on sectoral dairy relative imports is presented in Table 8, Table 9, Table 10, Table 11, Table 12 and Table 13 using the SUR model, where we consider the impact on sectoral trade with the world in relation to Australia and New Zealand combined in a single panel.

### 6.1. The Effects of Relative Imports Prices

Dairy products of Australia and New Zealand have an obvious comparative advantage in the Chinese market, and the fluctuation of their dairy prices has a great impact on China’s global imports. When the Chinese dairy import price relative to Australia or New Zealand $\ln\frac{P_{ijt}^{China}}{P_{imt}^{China}}$ increases, the global relative importation of Chinese dairy products will drop significantly. Dairy products are a daily necessity in consumer goods, but we have observed that China’s relative price elasticity in relation to imports from Australia and New Zealand is generally greater than 1 in all sectors of dairy products, meaning that the impact of these two countries on China’s relative imports of dairy products in the world was enormous. Compared with all sectors, New Zealand’s impact on China’s relative global dairy imports $\ln\frac{M_{ijt}^{China}}{M_{imt}^{China}}$ was significantly higher than that of Australia, especially in such sectors as liquid milk and cream with a fat content of less than 6% (HS040110 and HS040120 in Table 8), concentrated milk and cream containing added sugar with a fat content exceeding 1.5% (HS040229 in Table 9, milk and cream, containing added sugar or other sweetening matter (HS040299 in Table 9), yogurt (HS040310 in Table 10), whey (HS040410 in Table 11), butter and other fats and oils derived from milk (HS040500 in Table 12), cheese, blue-veined (HS040640 in Table 13). The impact of Australia on China’s relative global imports was also reflected in different sectors, but the magnitude of its influence was slightly below that of New Zealand, which indirectly indicates the important influence and status of New Zealand in these sectors on the global relative importation of Chinese dairy products.

### 6.2. The Influence of China’s Joining the WTO

Before China’s entry into the WTO in 2001, China imported fewer dairy products because of the restriction of the national economy and the people’s eating habits, as well as the imposition of high tariffs on dairy products. After 2001, thanks to the most-favored-nation treatment and the significant reduction in import tariffs on dairy products, the demand for domestic dairy products increased greatly, and the import volume and import quantity increased correspondingly.

We see that since China’s entry into the WTO, New Zealand has had a major impact on China’s relevant global imports, which were reflected in sectors including those for milk and cream, non-concentrated, with fat content more than 1% and 6% (HS040120 and HS040130 in Table 8), some concentrated milk and cream sectors (HS0402 in Table 9) such as concentrated milk and cream with a fat content (HS040210 and HS040221), Butter, curdled milk and cream, yogurt (HS0403), butter and other fats derived from milk (HS040510 in Table 12), fresh cheese not fermented and curd (HS040610 in Table 13) and cheese not grated, powdered or processed (HS040690 in Table 13), which are indirect indications of New Zealand’s competitive advantage in China in these sectors. We also find that after China’s entry into the WTO, there is a positive effect on China’s global imports of whey (HS040410 in Table 11). New Zealand has experienced quality problems in this sector and, although it has recovered its reputation in time after taking corrective action, the Chinese market has increased its imports from the rest of the world in whey. In other words, the impact from New Zealand in this sector has been greatly reduced. After China’s entry into the WTO, the relative impact of Australian dairy imports was not as obvious as those of New Zealand, and the impact of Australia on China’s global relative imports was also reflected in dry dairy products such as milk and cream, concentrated, with a fat content of less than 1.5% (HS040210 in Table 9). The largest demand for dairy imports from Chinese consumers is for infant milk powder. After China’s entry into the WTO, this sector experienced huge demand in the global market. Australia and New Zealand have more than a one-third share of the Chinese market in this sector, and they have a great impact on China’s global relative imports here.

### 6.3. The Influence of the China Melamine Milk Scandal

Before 2008, China’s dairy imports increased gradually as a result of its entry into the WTO and a drop in tariffs on dairy imports (shown in Figure 1). But after 2008, China’s dairy imports rose sharply, largely because of the 2008 melamine milk scandal. The 2008 melamine milk incident triggered kidney stones and threatened the lives of infants, but the Sanlu Group was evasive after the incident, which left Chinese consumers extremely dissatisfied and led to a crisis of confidence in the dairy industry. After the melamine incident, the Chinese government greatly increased its supervision of food products. However, domestic consumers still lack confidence in the quality of domestic dairy products and often prefer imported products, which has caused an overall increase in imports of dairy products to China after 2008. China’s domestic supply of liquid milk and infant formula has also been inadequate, spurring its global demand for imports.

We observe that the melamine incident had a greater impact on China’s global imports relative to Australia, especially regarding liquid dairy products such as liquid milk and cream with a fat content of more than 1% but less than 6% (HS040120) and with a fat content more than 6% (HS040130 in Table 8) in sector HS0401, as well as dry dairy products such as butter and other fats derived from milk (HS040590 in Table 12). New Zealand’s share of dairy products in the Chinese market was generally higher than Australia’s after the melamine milk scandal of 2008. After China and New Zealand agreed on an FTA, as tariffs fell, the import status of New Zealand dairy products in the Chinese market greatly increased and became firmly established. We observe that New Zealand had a clear impact on China’s global imports of both main liquid and dry dairy products and this is reflected in the sectors with a high market share in China. Compared with New Zealand, Australia has a slightly smaller market share in China (shown in Table 4), but for sectors such as whey and natural milk at HS040410 and HS040490 in Table 11, even though these were New Zealand’s most comparatively advantageous sectors, China’s global imports relative to Australia increased significantly and China’s global imports relative to New Zealand were greater, meaning that China bought more whey and natural milk from there than from any other country.

### 6.4. The Effects of the Milk Quality Scandal of New Zealand

A crisis of trust in China’s dairy industry, spreading even to the food industry as a whole was triggered by the melamine incidents in China, Chinese consumers had an unprecedented enthusiasm for imported dairy products from abroad, especially baby milk powder. However, New Zealand dairy products were found three times in the same month in 2013 to contain abnormal levels of whey protein nitrate; in addition, some of the whey protein was contaminated with Botox, all of which caused great panic among Chinese consumers. In a number of sectors, we have seen the impact of the New Zealand dairy scandal on China’s global imports from that country, for example, in liquid milk and cream, non-concentrated, with a fat content exceeding 10% (HS040150 in Table 8) and in whey (HS040410 in Table 11). The increase in China’s global imports from Australia was reflected in some dry dairy sectors such as milk and cream, concentrated or containing added sugar (HS040299 in Table 9), whey (HS040410 in Table 11), butter-related products (HS0405 including HS040510, HS040520, HS040590 in Table 12). It seems that the New Zealand milk scandal had little impact on China’s global imports of dry dairy products including cheese (HS0406 in Table 13). This implies that on the one hand New Zealand’s milk powder is still dominant in China, but on the other hand, the Chinese consumers preferred Australian dry dairy products. New Zealand’s cheese does not have a high market share in China and the proportion of cheese in Chinese food and beverages is low, in any case, so the relative impact on China’s global imports was not significant.

### 6.5. The Effects of the China-Australia FTA

In 2015, China and Australia signed a free trade agreement, under which China’s imports of dairy products from Australia were cut off [32]. Australian dairy production and exports are among the highest in the world, and the country is bound to become an important source of dairy products for China. But China imports dairy products from many sources, and the proportion of dairy products imported from Australia has not increased significantly either in the Chinese market or in the global market (Table 3 and Table 4). Compared with New Zealand, which also signed a free-trade agreement with China, Australia’s dairy products are significantly underrepresented in the Chinese market. Since the signing of the FTA between China and Australia after 2015, the proportion of dairy products imported from Australia to the world in 2017 was 7.8 percent, down from 8.49 in 2015, indicating that though China’s imports of dairy products from Australia might be expected to increase as tariffs fall, overall, there was still a downward trend. In other words, China imported a larger base of dairy products from the world (shown in Figure 1). Table 4 shows that China’s imports at sector HS0402 from Australia increased slightly after 2015. In Table 9, we found that milk and cream concentrated or containing added sugar (HS0402) imports from Australia, especially concentrated milk and cream containing added sugar, other than in solid forms (HS040299) have only a slight impact on China’s relative imports. Although the proportion of Australian liquid dairy (HS0401) in the Chinese market is not as high as that in New Zealand, we found that the China-Australia FTA in the HS040150 sector also has a negative impact on China’s global imports outside New Zealand (Table 8).

### 6.6. The Food Safety Law in China Dairy Imports

After the melamine milk scandal in 2008, the Chinese government, as noted above, issued a series of laws and regulations to supervise dairy product quality, signaling that the safe supervision model for food in China had changed from multiagency coordination to centralization [31]. After the enactment of China’s Food Safety Law, China’s food production and testing became more and more stringent, and dairy manufacturers and consumers’ requirements for dairy products also increased. As a result, China’s imports have become more and more diversified. We find that $D^{FSL\_China}$ has a positive impact on China’s global imports from Australia in the top three sectors (shown in Table 2), such as liquid milk and cream not containing sugar (HS0401 in Table 8), milk and cream concentrated or containing added sugar (HS0402 in Table 9) and whey and products consisting of natural milk constituents (HS0404 in Table 11). China imports a smaller proportion of dairy products from Australia than from New Zealand (see Table 4), Australia has played a catalytic role in China’s related global imports in these three sectors, especially in sectors such as milk and cream, not concentrated and not containing sugar, with a fat contents of more than 1% and less than 6% (HS040120) and with a fat content exceeding 6% (HS040130), milk and cream concentrated or containing added sugar, with a fat content exceeding 1.5% (HS040221 in Table 9) and whey (HS040410 in Table 11). The enforcement of Chinese food laws and the global demand for whey (HS040410) have greatly increased since the New Zealand whey protein scandal. Australia has indirectly promoted the impact of China’s whey on global imports. In contrast, New Zealand whey (HS040410) has had a stronger impact on China’s global relative imports, with a higher impact coefficient than other sectors, at 38.654. Although $D^{FSL\_China}$ has had a positive impact on China’s relative global imports, we have also found that China’s global imports of natural milk excluding whey (HS040490) have declined in relation to Australia, meaning that China puts more stringent controls on imports of such products.

In summary, we have found that, with China’s entry into the WTO, the import ratio of dairy products has increased significantly. Australia and New Zealand’s share of global imports in the Chinese market reached its highest, 72.52%, in 2010 (shown in Table 3), and their share in the HS0402 sector has consistently exceeded 30% of China’s total imports. New Zealand is China’s largest source of dairy products. In particular, it has the highest proportion in the HS0402, HS0401, HS0404 sectors. The cost of China’s imports relative to New Zealand on its global imports has a greater effect on its relative imports. New Zealand has a greater impact on China’s imports from the rest of the world. The emergence of the Chinese dairy scandal and the signing of the FTAs with New Zealand and Australia have greatly enhanced the market position of these two countries in China, especially New Zealand dairy products in their dominant sectors. But the subsequent whey scandal in New Zealand has also led to a sharp drop in Chinese imports in this sector, directly affecting China’s global relative import demand. In the wake of the melamine milk scandal in China, the inspection and supervision of dairy production and quality have become more stringent. While Australia and New Zealand remain important sources of solid and liquid dairy products for China, China’s global demand for their dominant dairy sectors remains significantly higher. It is worth mentioning that, China still favors New Zealand products and the total amount of dairy products imported from New Zealand has been increasing year by year, but when there are continuous problems in the quality of products, it has a lasting impact on China’s demand for imports from other milk-exporting countries.

## 7. Conclusions

Since China’s entry into the WTO in 2001, more than half of China’s global dairy imports have come from Australia and New Zealand, sharply increased by the domestic melamine milk scandal in 2008. We investigated China’s dairy imports in all dairy sectors in relation to its main dairy sources or FTA zones: Australia and New Zealand. Our emphasis was on examining the impact of the New Zealand whey scandal, the FTAs with Australia and New Zealand on China’s global relative dairy imports, in the context of China’s food safety laws after the melamine milk scandal.

We found that China’s import demand for dairy products increased year by year after its entry into the WTO, especially after the 2008 melamine milk scandal. China has the highest proportion of imports from three sectors. namely liquid dairy products, solid dairy products, and whey in sectors HS0402, HS0401, and HS0404 respectively. New Zealand has a higher import share of dairy products in China than Australia has, and its TC generally remains at 1. Its RCA is generally higher than that of Australia, which means that New Zealand’s dairy products are the most competitive in the Chinese market. New Zealand has had the greater impact on China’s global imports, especially in its dominant sector HS0402. The impact on China’s global relative imports has become most apparent with products including milk and cream, not concentrated, with a fat content of more than 1% and 6% (HS040120 and HS040130), and some milk and cream concentrated sectors (HS0402), such as concentrated milk and cream with a high fat content (HS040210 and HS040221), butter, curdled milk and cream, yogurt (HS0403), butter and other fats derived from milk (HS040510), fresh cheese not fermented and curd (HS040610) and cheese not grated, powdered or processed (HS040690). China’s global dairy imports dramatically increased in the wake of the 2008 melamine milk scandal. New Zealand’s dairy products have a significant impact on China’s imports from other regions, due to their clear comparative advantage and the establishment of an FTA with China. Although Australia and China also established an FTA, because Australia’s comparative advantage in China is far below that of New Zealand in dairy products, the impact of the China-Australia free trade zone on China’s relative global imports is reflected only in China’s highest demand sector, milk powder or solid dairy products in sector HS0402.

We also found that New Zealand dairy imports fell in China after the 2013 New Zealand whey scandal. A spate of dairy problems in New Zealand had a negative impact on both Chinese importers and consumers, leading to a significant increase in this sector of China’s imports from the rest of the world. It is worth mentioning that, due to the proficient implementation and clear credibility of its timely corrective actions, the New Zealand whey protein scandal did not tarnish the country’s dominant competitive position in the Chinese market—its TC for dairy products remained at 1 in all sectors. Although New Zealand’s RCA index in sectors HS0403, HS0405, HS0406 was less than 1, it was still higher than Australia’s. Chinese consumers were shocked by the 2013 New Zealand dairy product scandal while China’s quality inspection of dairy products had tended to tighten as far back as 2008. We also observed a sharp fall in New Zealand’s RCA index in the HS0404 sector after 2013 and the short-term negative impact of this on New Zealand’s overall imports. We find that the domestic dairy scandal in China had a huge impact on Chinese dairy imports from Australia and New Zealand and even on global relative imports, and this continues to this day. The New Zealand dairy quality scandal also had a short-term impact on the global demand of the Chinese dairy market and had a stimulating effect on China’s global demand specifically in the HS0404 sector.

New Zealand and Australia remain important sources of dairy imports for China; after all, they have more than 60% of the Chinese market. However, if New Zealand and Australia are to maintain their existing competitive position in the Chinese market, the quality and reputation of their products will be important determinants. With the improvement in China’s domestic food safety legislation, the inspection of dairy production and imports is becoming more and more stringent. We find that, although New Zealand and Australia have obvious comparative advantages in the Chinese market, China’s relative imports from the rest of the world are also rising in these sectors. China’s imports of dairy products in the HS0404 sector from the rest of the world have risen significantly, especially in consequence of the New Zealand whey protein scandal. It is not easy for New Zealand and Australia to maintain a sustainable competitive position in China and the world dairy market. For China’s dairy industry, there is great demand in the domestic market, the supervision of the dairy industry is being strengthened, thus gradually improving the international competitiveness of domestic enterprise products, and the level of imports from abroad will certainly rise. All these factors will make it more difficult for the world’s leading milk producers to maintain their competitiveness in the Chinese market.

All dairy products in the six sectors classified by the UN common trade scheme were considered in this study as we analyzed comparatively the role of Australia and New Zealand in the Chinese market. However, due to the data availability, some results cannot be calculated. For instance, the levels of exports or imports from Australia and New Zealand to China are unavailable in some years for some sections, such as milk and cream, not concentrated and with a fat contents of more than 6% and less than 10% (HS040140) for both countries from 1992 to 2010 and butter and other fats and oils derived from milk (HS040500), butter and other fats and oils derived from milk (HS 040590) for both countries after 1996 etc. As a result, the effects of both Australia and New Zealand on these sections of China’s cannot be calculated very accurately. In addition, direct methods are not always feasible to describe a policy, and this can make even the available information on food safety law (FSL) difficult to interpret. China has formulated a series of laws, regulations and standards to exert strict dairy product safety and quarantine measures on imported dairy products. In the present research, food safety law is used as a proxy for China’s dairy safety supervision.

It would be valuable for further studies to investigate China’s dairy imports in relation not only to Australia and New Zealand, but also from other main dairy exporters such as France, the Netherlands and the U.S. Furthermore, it would be worthwhile in further research to consider more dairy safety factors in relation to dairy products, using some alternative techniques to explain the data and produce robust results.

## Figures and Tables

**Table 1:** Chinese dairy imports and exports (Unit: USD in Millions).

	Harmonized System (HS)									
	HS 04 Dairy Products									
	1992	1995	2000	2003	2005	2008	2010	2013	2015	2017
Exports	158.8	162.2	187.9	221.6	267.4	872.8	404.6	544.8	606.1	588.2
Imports	68.8	63.6	217.9	349.8	461.8	621.0	2000.0	5245.2	3303.4	5070.0
Net Export	90.1	98.6	−30.0	−128.3	−194.4	−251.8	−1595.5	−4700.4	−2697.4	−4481.7

Note: Original Data were from UN ComTrade database 2018. Results were calculated by authors.

**Table 2:** China dairy imports and exports at sectors (Unit: USD in Millions).

		Harmonized System (HS)								
		HS 04 Dairy Products								
			1992	1995	2000	2005	2008	2010	2015	2017
Sector 0401	Milk and cream, not concentrated	EX	15.7	17.1	20.1	22.6	29.6	16.0	24.1	20.7
		IM	6.6	5.4	8.9	5.8	12.4	28.2	485.1	879.4
		Net EX	9.1	11.7	11.2	16.8	17.2	−12.2	−461.0	−858.7
Sector 0402	Milk and cream, concentrated	EX	5.9	9.1	27.9	55.9	248.4	15.3	15.4	14.3
		IM	34.1	29.0	115.7	235.4	401.4	1395.8	1529.1	2214.4
		Net EX	−28.2	−19.9	−87.8	−179.5	−153.0	−1380.5	−1513.7	−2200.2
Sector 0403	Buttermilk, curdled milk and cream, yogurt	EX	0.2	0.4	0.2	0.7	1.7	1.1	0.6	3.7
		IM	0.5	0.9	1.5	1.4	2.9	4.2	27.9	66.8
		Net EX	−0.4	−0.5	−1.3	−0.7	−1.1	−3.1	−27.4	−63.1
Sector 0404	Whey and product consisting of natural milk constituents	EX	0.3	0.2	0.3	0.5	4.8	0.8	0.1	0.4
		IM	15.8	19.4	80.0	157.4	312.0	344.8	524.3	666.1
		Net EX	−15.5	−19.2	−79.7	−156.9	−307.2	−344.0	−524.2	−665.7
Sector 0405	Butter and other fats and oils derived from milk; dairy spread	EX	0.1	0.2	0.4	0.1	17.2	9.7	4.0	7.6
		IM	3.0	1.3	4.7	31.8	59.2	91.4	265.5	499.5
		Net EX	−2.9	−1.1	−4.4	−31.7	−42.1	−81.7	−261.5	−491.9
Sector 0406	Cheese and curd	EX	0.1	0.3	1.2	1.9	1.5	0.9	1.0	1.2
		IM	1.5	2.1	3.9	26.4	53.8	105.4	348.1	497.5
		Net EX	−1.5	−1.8	−2.7	−24.5	−52.3	−104.5	−347.1	−496.3

Note: Original Data were from UN ComTrade database 2018 [26]. Results were calculated by authors.

**Table 3:** The amount and percentage of China dairy imports from Australia and New Zealand (Unit: USD in millions).

Year	Harmonized System (HS)					
	HS 04 Dairy Products					
	Australia		New Zealand		Sum	
	Value	% in World Dairy Imports	Value	% in World Dairy Imports	Value	% in World Dairy Imports
1992	4.09	6.64%	6.92	11.23%	11.01	17.64%
1995	5.07	8.79%	6.39	11.08%	11.46	19.79%
2000	27.34	12.76%	85.69	39.99%	113.03	52.76%
2005	48.76	10.67%	221.91	48.56%	270.67	59.67%
2008	126.35	14.48%	321.11	36.80%	447.46	51.27%
2010	127.74	6.52%	1299.92	66.35%	1427.66	72.52%
2015	263.53	8.49%	1749.68	56.37%	2013.21	64.49%
2017	369.28	7.80%	2771.96	58.55%	3141.24	66.80%

Note: Original Data were from UN ComTrade database 2018 [26]. Results were calculated by authors.

**Table 4:** Share of Australia and New Zealand in dairy market of China.

Code	Sector	Countries	Harmonized System (HS)						
			HS 04 Dairy Products						
			1992	1995	2000	2005	2010	2015	2017
HS0401	Milk and cream, not concentrated	Australia	0.49%	1.51%	2.47%	0.15%	0.07%	1.96%	1.36%
		New Zealand	0.03%	0.27%	0.67%	0.70%	0.79%	3.47%	7.45%
HS0402	Milk and cream, concentrated	Australia	3.09%	2.56%	6.17%	5.72%	4.36%	3.27%	3.77%
		New Zealand	3.34%	7.67%	35.56%	39.11%	56.84%	36.25%	32.57%
HS0403	Buttermilk, curdled milk and cream, yogurt	Australia	0.03%	0.46%	0.07%	0.07%	0.05%	0.06%	0.04%
		New Zealand	0.04%	0.06%	0.04%	0.04%	0.01%	0.29%	0.21%
HS0404	Whey and product consisting of natural milk constituents	Australia	1.76%	1.56%	3.23%	2.38%	0.47%	0.39%	0.19%
		New Zealand	5.78%	1.25%	0.47%	1.61%	1.04%	0.44%	0.28%
HS0405	Butter and other fats and oils derived from milk; dairy spread	Australia	0.14%	0.46%	0.08%	0.53%	0.36%	0.34%	0.21%
		New Zealand	0.58%	0.59%	1.90%	4.11%	3.67%	6.44%	8.21%
HS0406	Cheese and curd	Australia	0.43%	1.40%	0.52%	1.71%	1.07%	1.96%	1.72%
		New Zealand	0.30%	0.04%	0.68%	2.51%	2.53%	4.90%	4.82%

Note: Original Data were from UN ComTrade database 2018 [26]. Results were calculated by authors.

**Table 5:** Competition Index of Australia and New Zealand in China Market (competition index $=\frac{X_{i}-M_{i}}{X_{i}+M_{i}}$, $X_{i}$ means country i exports to China, $M_{i}$ means country i imports from China.).

Code	Sector	Country	1992	1995	2000	2005	2010	2015	2017
HS04	Dairy product	Australia	0.834	0.818	0.943	0.924	0.934	0.880	0.916
		New Zealand	0.996	0.985	1.000	1.000	0.999	0.998	0.999
HS0401	Milk and cream, not concentrated	Australia	1.000	1.000	1.000	0.867	1.000	1.000	1.000
		New Zealand	1.000	1.000	---	1.000	1.000	1.000	1.000
HS040110	Fat content ≤1%	Australia	---	1.000	1.000	0.829	1.000	1.000	1.000
		New Zealand	---	1.000	---	---	1.000	1.000	1.000
HS040120	Fat content >1% and ≤6%	Australia	1.000	1.000	1.000	0.810	1.000	1.000	1.000
		New Zealand	---	1.000	1.000	1.000	1.000	1.000	1.000
HS040130	Fat content >6%	Australia	1.000	1.000	1.000	1.000	1.000	1.000	1.000
		New Zealand	---	1.000	1.000	1.000	1.000	---	---
HS040140	Fat content >6% and ≤10%	Australia	---	---	---	---	---	1.000	1.000
		New Zealand	---	---	---	---	---	1.000	1.000
HS040150	Fat content >10%	Australia	---	---	---	---	---	1.000	1.000
		New Zealand	---	---	---	---	---	---	1.000
HS0402	Milk and cream, concentrated	Australia	1.000	1.000	1.000	0.991	0.998	0.998	1.000
		New Zealand	1.000	1.000	1.000	1.000	1.000	1.000	1.000
HS040210	Fat content ≤1.5%	Australia	---	---	1.000	1.000	1.000	1.000	1.000
		New Zealand	1.000	1.000	1.000	1.000	1.000	1.000	1.000
HS040221	Not containing added sugar, fat content >1.5%	Australia	1.000	1.000	1.000	1.000	1.000	1.000	1.000
		New Zealand	1.000	1.000	1.000	1.000	1.000	1.000	1.000
HS040229	Containing added sugar, fat content >1.5%	Australia	1.000	1.000	1.000	1.000	1.000	0.996	1.000
		New Zealand	1.000	1.000	1.000	1.000	0.996	1.000	1.000
HS040291	Not containing added sugar, other than in solid forms	Australia	---	1.000	1.000	0.886	---	1.000	1.000
		New Zealand	---	---	---	---	---	---	1.000
HS040299	Containing added sugar, other than in solid forms	Australia	---	1.000	1.000	1.000	0.703	0.726	0.990
		New Zealand	---	---	---	1.000	1.000	1.000	0.999
HS0403	Buttermilk, curdled milk and cream, yogurt	Australia	1.000	1.000	1.000	0.625	1.000	1.000	1.000
		New Zealand	1.000	1.000	1.000	1.000	1.000	1.000	1.000
HS040310	Yogurt	Australia	---	1.000	1.000	1.000	1.000	1.000	1.000
		New Zealand	---	1.000	1.000	1.000	1.000	1.000	1.000
HS040390	Buttermilk, curdled milk and cream	Australia	1.000	---	1.000	0.138	1.000	1.000	1.000
		New Zealand	1.000	1.000	1.000	1.000	1.000	1.000	1.000
HS0404	Whey and product consisting of natural milk constituents	Australia	1.000	1.000	1.000	1.000	1.000	0.996	1.000
		New Zealand	1.000	1.000	1.000	1.000	1.000	1.000	1.000
HS040410	Whey	Australia	1.000	1.000	1.000	1.000	1.000	0.996	1.000
		New Zealand	1.000	1.000	1.000	1.000	1.000	1.000	1.000
HS040490	Natural milk (excluding whey)	Australia	1.000	---	1.000	1.000	---	----	1.000
		New Zealand	1.000	1.000	1.000	1.000	1.000	1.000	1.000
HS0405	Butter and other fats and oils derived from milk; dairy spread	Australia	1.000	1.000	1.000	1.000	1.000	1.000	1.000
		New Zealand	1.000	1.000	1.000	1.000	1.000	---	1.000
HS040500	Butter and other fats and oils derived from milk	Australia	1.000	1.000	--	---	--	--	---
		New Zealand	1.000	1.000	--	---	--	--	---
HS040510	Derived from milk, butter	Australia	--	---	1.000	1.000	1.000	1.000	1.000
		New Zealand	--	---	1.000	1.000	1.000	1.000	1.000
HS040520	Dairy spreads	Australia	--	---	--	1.000	--	--	1.000
		New Zealand	--	---	--	--	--	--	--
HS040590	Fats and oils from derived from milk	Australia	--	--	--	--	--	--	--
		New Zealand	--	--	1.000	1.000	1.000	1.000	1.000
HS0406	Cheese and curd	Australia	0.961	1.000	1.000	1.000	0.979	1.000	1.000
		New Zealand	--	0.558	1.000	1.000	1.000	1.000	1.000
HS040610	Fresh cheese, not fermented and curd	Australia	1.000	1.000	1.000	1.000	1.000	1.000	1.000
		New Zealand	--	---	1.000	1.000	1.000	1.000	1.000
HS040620	Cheese of all kinds, grated or powdered	Australia	1.000	1.000	1.000	1.000	1.000	1.000	1.000
		New Zealand	--	1.000	1.000	1.000	1.000	1.000	1.000
HS040630	Cheese, processed	Australia	1.000	1.000	1.000	1.000	1.000	1.000	1.000
		New Zealand	---	1.000	1.000	1.000	1.000	1.000	1.000
HS040640	Cheese, blue-veined	Australia	---	---	---	1.000	1.000	---	---
		New Zealand	---	---	---	1.000	1.000	---	---
HS040690	Cheese (not grated, powdered or processed)	Australia	0.942	1.000	1.000	1.000	0.958	1.000	1.000
		New Zealand	---	0.974	1.000	1.000	1.000	1.000	1.000

Note: (1). Original data is from the UN ComTrade database 2018 [26], and results are calculated by the authors. (2). In the consensus of the competitiveness index, the statistical result is 1 or −1 due to the lack of import and export data, or the exports or imports alone.

**Table 6:** Revealed Competitive Advantages (RCA) in the Dairy Trading of Australia and New Zealand in China market. (Revealed comparative advantage index ${\mathrm{RCA}}_{i}=\frac{X_{i}/X}{X_{wi}/X_{w}}$, $X_{i},\;X_{wi}$ means dairy export of country i to China and the world, $X,X_{w}$ means the total export of country i to China and the world. When the RCA is calculated here, added value is used instead of total exports in order to avoid bias.)

Code	Sector	Countries	1992	1995	2000	2005	2010	2015	2017
HS04	Dairy product	Australia	0.001	---	0.007	0.003	0.002	0.004	0.006
		New Zealand	0.266	0.152	0.785	1.138	1.582	1.186	1.302
HS0401	Milk and cream, not concentrated	Australia	0.126	0.829	0.962	0.056	0.090	1.097	1.213
		New Zealand	---	0.333	0.174	1.046	1.125	3.002	3.230
HS040110	Fat content ≤1%	Australia	---	6.783	0.939	0.203	0.102	1.185	2.100
		New Zealand	---	0.050	---	---	1.377	2.162	2.035
HS040120	Fat content >1% and ≤6%	Australia	0.098	0.448	1.268	0.040	0.105	1.178	1.169
		New Zealand	---	0.902	0.202	0.193	0.265	2.645	3.039
HS040130	Fat content >6%	Australia	0.250	0.791	0.057	0.036	0.044	---	---
		New Zealand	---	0.032	0.124	2.707	2.599	---	---
HS040140	Fat content >6% and ≤10%	Australia	---	---	---	---	---	0.920	0.779
		New Zealand	---	---	---	---	---	3.351	2.877
HS040150	Fat content >10%	Australia	---	---	---	---	---	0.485	0.662
		New Zealand	---	---	---	---	---	---	3.359
HS0402	Milk and cream, concentrated	Australia	0.008	0.026	0.369	0.237	0.426	0.361	0.991
		New Zealand	0.198	0.224	1.553	1.885	2.469	1.486	1.585
HS040210	Fat content ≤1.5%	Australia	---	---	0.400	0.307	0.290	0.317	0.761
		New Zealand	0.065	0.154	1.113	1.812	1.336	1.793	1.448
HS040221	Not containing added sugar, fat content >1.5%	Australia	0.016	0.056	0.376	0.174	0.589	0.476	1.443
		New Zealand	0.253	0.246	1.796	1.929	2.846	1.407	1.597
HS040229	Containing added sugar, fat content >1.5%	Australia	0.026	0.093	0.016	0.182	0.672	0.922	1.945
		New Zealand	0.474	0.496	1.107	1.403	3.424	0.999	4.046
HS040291	Not containing added sugar, other than in solid forms	Australia	---	0.015	0.002	0.474	---	0.187	0.214
		New Zealand	---	---	---	---	---	---	0.451
HS040299	Containing added sugar, other than in solid forms	Australia	---	0.055	0.386	---	0.060	0.081	0.948
		New Zealand	---	---	---	0.022	---	---	4.428
HS0403	Buttermilk, curdled milk and cream, yogurt	Australia	0.005	0.001	0.031	0.063	0.094	0.063	0.109
		New Zealand	0.039	0.685	0.140	0.087	0.574	0.474	0.405
HS040310	Yogurt	Australia	---	0.005	0.167	0.195	0.209	0.097	0.136
		New Zealand	---	0.054	0.439	1.253	0.750	0.931	0.286
HS040390	Buttermilk, curdled milk and cream	Australia	0.005	---	0.006	0.025	0.045	0.036	0.050
		New Zealand	0.041	0.721	0.134	0.058	0.573	0.386	0.406
HS0404	Whey and product consisting of natural milk constituents	Australia	1.661	2.594	2.056	0.908	0.592	0.651	0.604
		New Zealand	22.417	1.277	0.780	0.497	0.638	0.162	0.183
HS040410	Whey	Australia	0.425	3.675	2.628	0.975	0.597	0.665	0.606
		New Zealand	3.232	2.649	3.302	8.245	6.323	0.952	1.025
HS040490	Natural milk (excluding whey)	Australia	4.443	---	0.232	0.348	---	---	0.509
		New Zealand	---	---	0.045	0.106	0.267	0.120	0.109
HS0405	Butter and other fats and oils derived from milk; dairy spread	Australia	0.012	0.105	0.031	0.048	0.071	0.214	0.438
		New Zealand	0.272	0.081	0.060	0.564	0.437	0.791	0.862
HS040500	Butter and other fats and oils derived from milk	Australia	0.012	0.105	---	---	---	---	---
		New Zealand	0.272	0.081	---	---	---	---	---
HS040510	Derived from milk, butter	Australia	---	---	0.061	0.103	0.087	0.317	0.622
		New Zealand	---	---	0.049	0.427	0.394	1.086	1.031
HS040520	Dairy spreads	Australia	---	---	---	0.015	---	---	0.007
		New Zealand	---	---	---	---	---	---	---
HS040590	Fats and oils from derived from milk	Australia	---	---	---	0.004	0.050	---	---
		New Zealand	---	---	0.083	0.899	0.506	0.448	0.643
HS0406	Cheese and curd	Australia	0.010	0.039	0.006	0.095	0.111	0.275	0.401
		New Zealand	---	0.007	0.038	0.290	0.424	0.804	0.802
0HS040610	Fresh cheese, not fermented and curd	Australia	0.037	0.225	0.001	0.049	0.125	0.297	0.502
		New Zealand	---	---	0.136	0.570	1.542	2.301	1.928
HS040620	Cheese of all kinds, grated or powdered	Australia	0.015	0.009	0.010	0.063	0.044	0.075	0.141
		New Zealand	---	---	0.146	1.721	1.397	1.813	1.530
HS040630	Cheese, processed	Australia	0.005	0.026	0.008	0.149	0.147	0.124	0.184
		New Zealand	---	0.024	0.020	0.172	0.412	0.789	0.916
HS040640	Cheese, blue-veined	Australia	---	---	---	0.021	0.020	---	---
		New Zealand	---	---	---	0.291	0.000	---	---
HS040690	Cheese (not grated, powdered or processed)	Australia	0.012	0.034	0.006	0.083	0.100	0.317	0.393
		New Zealand	---	0.006	0.006	0.045	0.028	0.146	0.188

Note: Original data is from UN ComTrade database, 2018 [26]; results are calculated by authors.

**Table 7:** Harmonized System Sectors Classification.

HS 04 Dairy Product	
HS0401;	Milk and Cream, not concentrated, not containing added sugar or other sweetening matter;
HS040110	Fat content, by weight, not exceeding 1%;
HS040120	Fat content, by weight, exceeding 1% but not exceeding 6%;
HS040130	Fat content, by weight, exceeding 1% exceeding 6%;
HS040140	Fat content, by weight, exceeding 6% but not exceeding 10%;
HS040150	Fat content, by weight, exceeding 10%;
HS0402	Milk and Cream, concentrated or containing added sugar or other sweetening matter;
HS040210	In powder, granules or other solid forms, of a fat content not exceeding 1.5% (by weight);
HS040221	Not containing added sugar or other sweetening matter, in powder, granules or other solid forms, of a fat content exceeding 1.5% (by weight);
HS040229	In powder, granules or other solid forms, of a fat content exceed 1.5% (by weight);
HS040291	Not containing added sugar or other sweetening matter, other than in powder granules or other solid forms;
HS040299	Other than in powder granules or other solid forms;
HS0403	Buttermilk, curdled milk and cream, yoghurt, kephir, fermented or acidified milk or cream, whether or not concentrated, containing added sugar, sweetening matter, flavoured or added fruit or cocoa;
HS040310	Yoghurt;
HS040390	Buttermilk, curdled milk or cream, kephir, fermented or acidified milk or cream;
HS0404	Whey and product consisting of natural milk constituents, whether or not containing added sugar or other sweetening matter, not elsewhere specified or included;
HS040410	Whey;
HS040490	Natural milk constituents (excluding whey);
HS0405	Butter and other fats and oils derived from milk; dairy spreads;
HS040500	Butter and other fats and oils derived from milk;
HS040510	Derived from milk, butter;
HS040520	Dairy spreads;
HS040590	Fats and oils derived from milk (other than butter or dairy spreads);
HS0406	Cheese and curd;
HS040610	Fresh cheese (including whey cheese), not fermented, and curd;
HS040620	Cheese of all kinds, grated or powdered;
HS040630	Cheese, processed (not grated or powered);
HS040640	Blue-veined and other cheese containing veins produced by penicillium roqueforti (not grated, powdered or processed);
HS040690	Cheese (not grated, powdered or processed);

Note: all information is from UN Comtrade database, 2018 [26] and compiled by authors.

**Table 8:** Impact of Australia and New Zealand Dairy Products on China Imports at Sector HS0401 Using the SUR Model.

	Variables	Impact Countries	Constant	$ln\frac{P_{ijt}^{China}}{P_{imt}^{China}}$	$D_{t}^{WTO}$	$D_{t}^{MMS\_China}$	$D_{t}^{MS\_NZ}$	$D_{t}^{FTA\_Aus}$	$D^{FSL\_China}$	*R* ^2^	*Observations*
Sector											
HS0401 Milk and cream, not concentrated, not containing added sugar		Australia	5.109 *** (3.47)	−2.635 *** (3.16)	−2.764 (1.15)	3.265 *** (3.34)	0.016 (0.00)	0.432 (0.11)	8.712 ** (2.04)	0.6276	26
		New Zealand	6.438 *** (3.22)	−4.773 ** (2.31)	−4.455 ** (1.98)	−1.351 ** (2.04)	1.356 (0.03)	−1.220 (0.02)	−0.532 (0.01)	0.6311	26
HS040110 Fat content ≤1%		Australia	9.294 *** (2.79)	−1.176 ** (2.38)	5.178 (1.60)	0.137 (0.04)	−0.474 (0.10)	0.967 (0.17)	−1.254 (0.19)	0.5619	26
		New Zealand	7.770 *** (3.57)	−4.900 *** (4.91)	−3.406 (1.05)	−3.203 ** (2.21)	48.406 (0.08)	−4.991 (0.06)	85.680 (0.11)	0.7672	26
HS040120 Fat content >1% and ≤6%		Australia	8.903 *** (2.36)	−1.815 *** (3.54)	−1.378 (0.96)	2.283 *** (3.05)	2.358 (1.00)	−0.262 (0.09)	5.266 (1.73)	0.5301	26
		New Zealand	3.492 *** (4.05)	−6.851 *** (5.96)	−6.411 ** (2.14)	2.077 (0.06)	6.940 (0.15)	−5.479 (0.10)	4.110 (0.07)	0.5708	26
HS040130 Fat content >6%		Australia	3.27 ** (2.31)	−1.85 ** (2.34)	5.71 (0.17)	3.191 * (1.76)	1.375 (0.25)	0.165 (0.43)	12.400 (2.15)	0.5593	26
		New Zealand	2.877 *** (2.63)	−2.705 *** (12.12)	−2.632 *** (3.64)	−1.203 *** (3.52)	0.556 (1.32)	0.155 (1.33)	1.668 (0.13)	0.7222	26
HS040140 Fat content >6% and ≤10%		Australia	---	---	---	---	---	---	---	---	---
		New Zealand	---	---	---	---	---	---	---	---	---
HS040150 Fat content >10%		Australia	6.139 *** (3.24)	−1.490 ** (2.16)	−2.331 (0.35)	2.135 (0.989)	0.435 (1.56)	−3.752 (1.22)	4.350 (1.45)	0.6928	20
		New Zealand	8.64 *** (9.37)	−3.485 *** (7.75)	−1.536 (0.75)	1.633 (0.763)	0.719 *** (13.21)	−0.780 *** (10.37)	2.545 (0.758)	0.5073	20

Note: * means significant at 10%, ** means significant at 5%, *** means significant at 1%. Original data is from UN Comtrade database, 2018 [26]; results are calculated by authors.

**Table 9:** Impact of Australia and New Zealand Dairy Products on China imports at Sector HS0402 Using the SUR Model.

	Variables	Impact Countries	Constant	$ln\frac{P_{ijt}^{China}}{P_{imt}^{China}}$	$D_{t}^{WTO}$	$D_{t}^{MMS\_China}$	$D_{t}^{MS\_NZ}$	$D_{t}^{FTA\_Aus}$	$D^{FSL\_China}$	*R* ^2^	*Observations*
Sector											
HS0402 Milk and cream, concentrated or containing added sugar		Australia	9.269 ** (2.63)	−1.654 ** (2.09)	−0.200 (1.46)	1.609 *** (3.17)	1.002 ** (2.09)	−1.708 *** (2.84)	17.011 (3.24)	0.7176	26
		New Zealand	6.582 (1.24)	−3.833 *** (3.18)	−4.331 *** (2.93)	−3.097 *** (3.06)	−0.069 (0.03)	0.073 (0.03)	−0.592 (0.19)	0.4385	26
HS040210 Fat content ≤1.5%		Australia	12.991 *** (2.59)	−0.735 *** (2.80)	−4.927 *** (3.69)	4.068 (0.88)	6.941 (1.05)	−6.744 (0.92)	6.487 (0.73)	0.6435	26
		New Zealand	20.778 * (177)	−1.214 *** (5.98)	−7.779 (2.68)	−1.428 ** (2.13)	0.491 (0.10)	0.293 (0.06)	0.173 (0.03)	0.7533	26
HS040221 Not containing added sugar Fat content >1.5%		Australia	14.305 ** (2.25)	−1.985 *** (5.22)	1.163 (0.14)	2.513 ** (2.40)	3.326 (0.25)	−1.780 (0.71)	38.408 (2.39)	0.5312	26
		New Zealand	10.437 ** (2.25)	−3.507 ** (2.18)	−2.814 *** (3.79)	−1.326 ** (2.03)	−0.078 (0.06)	−0.130 (0.10)	−0.404 (0.24)	0.5770	26
HS040229 Containing added sugar, fat content >1.5%		Australia	5.212 ** (2.04)	−2.220 ** (2.36)	1.904 (0.32)	12.464 (1.84)	273.851 (1.62)	−4.060 (2.71)	22.458 (1.04)	0.5535	26
		New Zealand	6.144 (1.61)	−5.234 *** (6.45)	−2.990 (0.73)	−1.043 ** (2.29)	−0.331 (0.06)	5.366 (0.96)	−0.287 (0.04)	0.6113	26
HS040291 Not containing added sugar, other than in solid forms		Australia	6.215 *** (3.11)	−0.801 *** (7.09)	−0.884 (0.10)	−0.764 (0.09)	8.552 (0.77)	−1.376 (0.85)	−0.964 (0.06)	0.7435	26
		New Zealand	3.568 ** (2.06)	−3.702 *** (3.87)	−3.039 (0.79)	2.742 (0.53)	1.908 *** (2.73)	0.51 (0.82)	28.840 (0.30)	0.7990	26
HS040299 Containing added sugar, other than in solid forms		Australia	6.933 (1.13)	−2.598 *** (4.35)	−0.807 (0.00)	−6.593 (0.03)	1.89 *** (3.68)	−1.42 (3.40)	7.426 (1.56)	0.8722	26
		New Zealand	5.301 *** (3.71)	−6.616 ** (2.32)	0.750 (0.04)	−2.405 *** (2.75)	−7.080 (0.94)	1.536 (1.43)	22.87 (0.536)	0.7325	26

Note: * means significant at 10%, ** means significant at 5%, *** means significant at 1%. Original data is from UN Comtrade database, 2018 [26]; results are calculated by authors.

**Table 10:** Impact of Australia and New Zealand Dairy Products on China imports at Sector HS0403 Using the SUR Model.

	Variables	Impact Countries	Constant	$ln\frac{P_{ijt}^{China}}{P_{imt}^{China}}$	$D_{t}^{WTO}$	$D_{t}^{MMS\_China}$	$D_{t}^{MS\_NZ}$	$D_{t}^{FTA\_Aus}$	$D^{FSL\_China}$	*R* ^2^	*Observations*
Sector											
HS0403 Buttermilk, curdled milk and cream, yogurt		Australia	16.583 (1.21)	−2.759 *** (3.10)	−6.304 (1.63)	1.874 (0.43)	10.771 (1.72)	6.024 (0.87)	3.091 (0.36)	0.6696	26
		New Zealand	34.583 ** (2.08)	−6.801 *** (5.87)	−2.245 *** (2.62)	3.990 (0.78)	−19.804 (0.85)	3.025 (0.12)	−48.870 (1.67)	0.7604	26
HS040310 Yogurt		Australia	70.147 ** (2.25)	−1.086 *** (3.97)	−3.426 (0.36)	11.280 (0.46)	−0.493 (0.01)	1.413 (0.28)	−4.051 (0.09)	0.5753	26
		New Zealand	13.678 ** (2.55)	−4.329 *** (5.83)	−2.538 ** (2.18)	9.414 (0.34)	−3.682 (0.78)	1.396 (0.41)	−47.205 (0.85)	0.7585	22
HS040390 Buttermilk, curdled milk and cream		Australia	28.176 * (1.84)	−1.787 ** (2.26)	1.101 (1.09)	4.021 (0.26)	−5.144 (0.27)	3.362 (0.01)	3.594 (0.81)	0.6579	26
		New Zealand	13.916 *** (2.98)	−3.210 *** (3.25)	−2.423 ** (2.56)	−8.376 (0.16)	−4.876 (0.66)	1.081 (0.13)	−108.876 (1.10)	0.6949	26

Note: * means significant at 10%, ** means significant at 5%, *** means significant at 1%. Original data is from UN Comtrade HS, 2018 [26]; results are calculated by authors.

**Table 11:** Impact of Australia and New Zealand Dairy Products on China imports at Sector HS0404 Using the SUR Model.

	Variables	Impact Countries	Constant	$ln\frac{P_{ijt}^{China}}{P_{imt}^{China}}$	$D_{t}^{WTO}$	$D_{t}^{MMS\_China}$	$D_{t}^{MS\_NZ}$	$D_{t}^{FTA\_Aus}$	$D^{FSL\_China}$	*R* ^2^	*Observations*
Sector											
HS0404 Whey and product consisting of natural milk constituents		Australia	10.579 ** (2.13)	−1.412 *** (5.12)	1.618 (0.44)	2.151 *** (5.83)	3.851 *** (4.78)	−8.403 (1.26)	12.856 ** (2.53)	0.9058	26
		New Zealand	7.039 *** (4.49)	−3.795 *** (4.04)	2.260* (1.90)	−3.625 ** (2.56)	36.034 (1.08)	−1.927 (0.30)	17.241 ** (2.37)	0.7249	26
HS040410 Whey		Australia	5.212 ** (2.40)	−1.092 *** (3.30)	1.905 (0.48)	2.209 *** (5.25)	1.110 *** (4.41)	−4.008 (0.54)	17.920 ** (2.26)	0.8953	26
		New Zealand	4.354 (1.46)	−4.682 *** (5.85)	3.348 ** (2.15)	−7.001 *** (2.70)	2.204 *** (3.11)	−1.315* (1.80)	38.654 *** (2.58)	0.6759	26
HS040490 Natural milk (excluding whey)		Australia	1.888 ** (2.01)	−1.143 *** (4.07)	12.383 (0.03)	1.041 *** (3.08)	−975.381 (1.47)	−5.211 (0.85)	−3.224 *** (17.24)	0.5576	26
		New Zealand	3.189 *** (7.45)	−3.859 *** (2.46)	0.989 (0.64)	−5.267 (1.10)	−33.745 (0.05)	2.211 (0.04)	−30.767 (0.03)	0.6416	19

Note: * means significant at 10%, ** means significant at 5%, *** means significant at 1%. Original data is from UN Comtrade database, 2018 [26]; results are calculated by authors.

**Table 12:** Impact of Australia and New Zealand Dairy Products on China imports at Sector HS0405 Using the SUR Model.

	Variables	Impact Countries	Constant	$ln\frac{P_{ijt}^{China}}{P_{imt}^{China}}$	$D_{t}^{WTO}$	$D_{t}^{MMS\_China}$	$D_{t}^{MS\_NZ}$	$D_{t}^{FTA\_Aus}$	$D^{FSL\_China}$	*R* ^2^	*Observations*
Sector											
HS0405 Butter and other fats and oils derived from milk; dairy spread		Australia	7.671 ** (2.07)	−1.305 *** (3.73)	2.646 (0.56)	3.266 (0.64)	2.227 (2.82)	−2.285 (0.30)	1.700 (0.17)	0.6161	26
		New Zealand	1.542 (1.44)	−5.854 *** (2.71)	−2.978 *** (3.08)	−0.104 ** (2.27)	−0.083 (0.05)	−0.022 (0.01)	−0.122 (0.06)	0.6675	26
HS040500 Butter and other fats and oils derived from milk		Australia	2.351 ** (2.19)	−1.435 ** (2.53)	---	---	---	---	---	---	6
		New Zealand	3.102 (1.52)	−2.324 *** (2.66)	---	---	---	---	---	---	6
HS040510 Derived from milk, butter		Australia	14.429 *** (6.63)	−0.679 *** (4.71)	6.783 (1.57)	−3.784 (0.96)	1.400 (2.20)	2.180 (0.36)	0.130 (0.02)	0.6443	22
		New Zealand	10.176 *** (3.13)	−1.806 *** (5.31)	−0.618 *** (3.27)	−0.112 ** (2.57)	−0.130 (0.48)	0.029 (0.10)	−0.130 (0.35)	0.5406	22
HS040520 Dairy spreads		Australia	9.377 *** (5.58)	−2.086 ** (2.29)	−2.974 (1.18)	−11.902 (0.93)	1.661 (4.74)	1.242 (0.27)	0.862 (0.66)	0.9123	22
		New Zealand	---	---	---	---	---	---	---	---	---
HS040590 Fats and oils derived from milk		Australia	8.752 *** (2.83)	−1.961 *** (5.08)	−6.085 (0.64)	1.385 ** (2.54)	3.737 (6.89)	---	---	0.8783	26
		New Zealand	3.464 (1.13)	−2.102 ** (2.08)	2.710 (0.53)	1.453 (0.82)	1.212 (1.23)	3.012 (0.83)	−0.035 (0.04)	0.6735	24

Note: * means significant at 10%, ** means significant at 5%, *** means significant at 1%. Original data is from UN Comtrade database, 2018 [26]; results are calculated by authors.

**Table 13:** Impact of Australia and New Zealand Dairy Products on China imports at Sector HS0406 Using the SUR Model.

	Variables	Impact Countries	Constant	$ln\frac{P_{ijt}^{China}}{P_{imt}^{China}}$	$D_{t}^{WTO}$	$D_{t}^{MMS\_China}$	$D_{t}^{MS\_NZ}$	$D_{t}^{FTA\_Aus}$	$D^{FSL\_China}$	*R* ^2^	*Observations*
Sector											
HS0406 Cheese and curd		Australia	1.745 (1.56)	−2.387 *** (2.89)	−0.207 (0.24)	0.567 (0.73)	−0.365 (0.35)	1.129 (1.00)	0.335 (0.24)	0.6184	26
		New Zealand	−4.021 *** (2.88)	−1.242 ** (2.07)	−6.353 (0.77)	−0.783 ** (2.10)	1.547 (0.13)	−0.310 (0.02)	0.602 * (1.89)	0.7606	26
HS040610 Fresh cheese, not fermented and curd		Australia	25.925 *** (3.25)	−2.418 *** (3.57)	4.997 (0.96)	−74.341 (0.12)	1.656 (0.38)	2.138 (0.03)	−21.676 (0.02)	0.6557	26
		New Zealand	26.804 ** (2.11)	−1.904 *** (5.09)	−5.138 ** (2.12)	−1.1397 ** (2.18)	−0.554 (0.06)	−0.107 (0.01)	−0.828 (0.07)	0.5854	26
HS040620 Cheese of all kinds, grated or powdered		Australia	15.917 *** (2.61)	−1.968 ** (2.31)	−5.917* (1.74)	10.432 (0.28)	1.129 (0.32)	6.259 (1.23)	25.582 (0.37)	0.6129	26
		New Zealand	11.551 *** (4.69)	−1.969 ** (2.19)	5.671 (0.54)	−1.834 (2.61)	0.162 (0.01)	−0.055 (0.22)	0.116 * (1.81)	0.5890	24
HS040630 Cheese, processed		Australia	−0.470 (1.13)	−5.262 ** (2.45)	2.724 (0.95)	−5.631* (1.91)	0.406 (0.10)	5.091 (1.11)	−1.343 (0.23)	0.5954	26
		New Zealand	−2.985 ** (2.07)	−2.013 *** (4.32)	2.540 (0.05)	4.322 (0.09)	7.518 (0.10)	8.177 (0.10)	0.730 * (1.79)	0.5386	26
HS040640 Cheese, blue-veined		Australia	−64.547 (1.08)	−5.547 ** (2.08)	1.003 (0.69)	−59.546 (0.37)	---	---	---	05722	6
		New Zealand	−24.992 ** (2.09)	−7.503 *** (6.11)	3.97* (1.81)	−10.526 (0.17)	---	---	---	0.6306	6
HS040690 Cheese (not grated, powdered or processed)		Australia	1.881 (1.63)	−1.206 *** (5.206)	0.141 (0.23)	−0.393 (0.56)	−0.397 (0.40)	0.314 (0.29)	−0.925 (0.69)	0.5253	26
		New Zealand	2.911 *** (2.82)	−2.369 *** (3.75)	−7.426 *** (4.73)	0.232 *** (3.12)	2.818 (0.31)	−0.499 (0.05)	4.192 (0.34)	0.5946	26

Note: * means significant at 10%, ** means significant at 5%, *** means significant at 1%. Original data is from UN Comtrade database, 2018 [26]; results are calculated by authors.
